# Developmental endothelial locus-1 as a potential biomarker for the incidence of acute exacerbation in patients with chronic obstructive pulmonary disease

**DOI:** 10.1186/s12931-021-01878-7

**Published:** 2021-11-20

**Authors:** Dong-Hyun Joo, Kyoung-Hee Lee, Chang-Hoon Lee, Jisu Woo, Jiyeon Kim, Seoung Ju Park, Chin Kook Rhee, Won-Yeon Lee, Dongil Park, Jae Seung Lee, Ki-Suck Jung, Kwang Ha Yoo, Chul-Gyu Yoo

**Affiliations:** 1 Division of Pulmonary and Critical Care Medicine, Department of Internal Medicine, Seoul National University Hospital, Seoul, 03080 Republic of Korea; 2 Department of Internal Medicine, Jeonbuk National University Medical School, Jeonju, Republic of Korea; 3 Division of Pulmonary, Allergy and Critical Care Medicine, Department of Internal Medicine, Seoul St. Mary’s Hospital, College of Medicine, The Catholic University of Korea, Seoul, Republic of Korea; 4 Department of Internal Medicine, Yonsei University Wonju College of Medicine, Wonju, Republic of Korea; 5 Division of Pulmonary and Critical Care Medicine, Department of Internal Medicine, Chungnam National University Hospital, Daejeon, Republic of Korea; 6 Department of Pulmonary and Critical Care Medicine, Asan Medical Center, University of Ulsan College of Medicine, Seoul, Republic of Korea; 7 Division of Pulmonary, Allergy and Critical Care Medicine, Department of Medicine, Hallym University Sacred Heart Hospital, Hallym University Medical School, Anyang, Republic of Korea; 8 Department of Internal Medicine, Division of Pulmonary and Allergy Medicine, Konkuk University School of Medicine, Seoul, Republic of Korea; 9 Department of Internal Medicine, Seoul National University College of Medicine, Seoul, Republic of Korea

**Keywords:** Chronic obstructive pulmonary disease, Developmental endothelial locus-1, Disease progression, Biomarkers, Animal disease models

## Abstract

**Background:**

Despite the high disease burden of chronic obstructive pulmonary disease (COPD) and risk of acute COPD exacerbation, few COPD biomarkers are available. As developmental endothelial locus-1 (DEL-1) has been proposed to possess beneficial effects, including anti-inflammatory effects, we hypothesized that DEL-1 could be a blood biomarker for COPD.

**Objective:**

To elucidate the role of plasma DEL-1 as a biomarker of COPD in terms of pathogenesis and for predicting acute exacerbation.

**Methods:**

Cigarette smoke extract (CSE) or saline was intratracheally administered to wild-type (WT) and DEL-1 knockout (KO) C57BL/6 mice. Subsequently, lung sections were obtained to quantify the degree of emphysema using the mean linear intercept (MLI). Additionally, plasma DEL-1 levels were compared between COPD and non-COPD participants recruited in ongoing prospective cohorts. Using negative binomial regression analysis, the association between the plasma DEL-1 level and subsequent acute exacerbation risk was evaluated in patients with COPD.

**Results:**

In the in vivo study, DEL-1 KO induced emphysema (KO saline vs. WT saline; *P* = 0.003) and augmented CSE-induced emphysema (KO CSE vs. WT CSE; *P* < 0.001) in 29 mice. Among 537 participants, patients with COPD presented plasma log (DEL-1) levels lower than non-COPD participants (*P* = 0.04), especially non-COPD never smokers (*P* = 0.019). During 1.2 ± 0.3 years, patients with COPD in the lowest quartile of Log(DEL-1) demonstrated an increased risk of subsequent acute exacerbation, compared with those in the highest quartile of Log(DEL-1) (adjusted incidence rate ratio, 3.64; 95% confidence interval, 1.03–12.9).

**Conclusion:**

Low DEL-1 levels are associated with COPD development and increased risk of subsequent COPD acute exacerbation. DEL-1 can be a useful biomarker in patients with COPD.

**Supplementary Information:**

The online version contains supplementary material available at 10.1186/s12931-021-01878-7.

## Background

Chronic obstructive pulmonary disease (COPD) is a common respiratory disease, with a reported worldwide prevalence of 3.9% in 2017 [1]*.* Although COPD is a chronic disease, a substantial number of patients experience acute exacerbation [2]*.* Acute exacerbation is known to result in a reduced quality of life [3]*,* disease progression [4]*,* and even premature mortality. Globally, COPD was responsible for 5.7% of all-cause mortality in 2017 [1], projected to increase to 7.8% in 2030 [5]*.* Despite the importance of this disease, limited advances have been achieved in its treatment. Obstacles to advances in COPD therapeutics include unclear pathogenesis that needs to be comprehensively clarified [6]*,* the complexity and heterogeneity of the disease [7]*,* and a lack of biomarkers that can be easily determined. Although the forced expiratory volume in one second (FEV1) had long been used as an indicator of COPD severity in Global Initiative for Obstructive Lung Disease (GOLD) [8], FEV1 only weakly correlated with symptoms and health status [9]. Therefore, GOLD introduced ABCD system including COPD assessment test and prior history of acute exacerbation [10], and the needs for other biomarkers increased, which led to investigations of various biomarkers. As the difficulty in obtaining specimens such as sputum or bronchoalveolar lavage fluid hinder application, blood biomarkers have been of great interest recently. Indeed, COPD is not only characterized by airflow limitation but also by systemic manifestations [11]. A few blood biomarkers linked to systemic inflammation such as C-reactive protein (CRP), interleukins (ILs), fibrinogen and tumor necrosis factor (TNF)-α have been identified [12]. However, although these biomarkers are associated with COPD outcomes including acute exacerbation [12], the relationship between airway inflammation, that is the key pathogenesis in acute exacerbation, and systemic inflammation is unclear [13, 14]. There are other blood biomarkers, including surfactant protein D (SP-D) [15] and club cell protein-16 (CC-16) [16], associated with acute exacerbation, however, those have also limitations as reliable biomarkers due to conflicting results [17] and small size study [16].

Developmental endothelial locus-1 (DEL-1), also known as epidermal growth factor (EGF)-like repeats and discoidin I-like domains 3 (EDIL3), is an endogenous protein that is produced particularly in the brain and lungs, and secreted by various cells so that it can be easily measured in serum or plasma [18]. Reportedly, DEL-1 demonstrates anti-inflammatory effects by inhibiting neutrophil recruitment and promoting inflammation clearance [19–21]*.* Airway and systemic inflammation are principal mechanisms of acute exacerbation of COPD [22]*.* Furthermore, DEL-1 has inhibitory effects on endothelial cell apoptosis [23]*,* as well as anti-fibrotic [24, 25], anti-aging [26], and anti-protease effects [27, 28], indicating that DEL-1 could have beneficial effects in COPD [28]. However, the majority of studies reporting the functions of DEL-1 were at the stage of mice or cells, and only a few studies investigated in humans the association between DEL-1 and humans diseases such as multiple sclerosis [29], periodontitis [30] and sepsis [31]. The effects of DEL-1 on COPD have not been studied in mice or humans. Therefore, we conducted the study in mice and humans to investigate whether DEL-1 could be a blood biomarker for the pathogenesis of COPD and for predicting acute exacerbation of COPD.

## Methods

### In vivo study

#### CSE preparation

Cigarette smoke extract (CSE) was prepared as previously described [32] and is detailed in Additional file 1.

#### Mice and intratracheal administration of CSE

Female 8-week-old C57BL/6 wild-type (WT) mice were purchased from Koatech Laboratory Animal Company (Pyeongtaek, Korea). DEL-1 knockout (KO) mice on a C57BL/6 genetic background were kindly donated by Dr. Eun Young Choi (Asan Medical Center, Seoul, Korea). C57BL/6 WT and DEL-1 KO mice were anesthetized and intratracheally administered saline or 100 μL CSE. CSE was administered once a week for 8 weeks (WT saline, n = 8; WT CSE, n = 8; KO saline, n = 5; KO CSE, n = 8). To isolate the lungs, the mice were killed on the day after the last CSE instillation. Animal experiments were approved by the Institutional Animal Care and Use Committee (number 17-0134-C1A0(2)) of Seoul National University Hospital, Seoul, Korea.

#### Reverse transcriptase-PCR (RT-PCR)

Total RNA was isolated from different tissues of WT and DEL-1 KO C57BL/6 mice. Total RNA was isolated using a RNeasy kit (Qiagen, Hilden, Germany). cDNA was synthesized using the Reverse Transcription System (Promega, Madison, WI, USA). PCR Master Mix (Applied Biosystems, Carlsbad, CA, USA) was used for amplification. The primers used in the study were as follows: mouse Del-1 primer (fwd: 5′-CCT GTG AGA TAA GCG AAG-3′, rev: 5′-GAG CTC GGT GAG TAG ATG-3′) and GAPDH (fwd: 5′-TCC CTC AAG ATT GTC AGC AAT G-3′, rev: 5′-AGA TCC ACA ACG GAT ACA TTG G-3′). PCR conditions were as follows: initial denaturation at 94 °C for 2 min; 40 cycles were performed for the mRNA levels of DEL-1 and GAPDH, each cycle with 20 s of denaturation at 94 °C, 10 s of annealing at 60 °C, and 30 s of extension at 72 °C, with a final dwell at 72 °C for 5 min.

#### Measurement of emphysema

The mean linear intercept (MLI) was measured to quantify emphysema as previously described [33] and is detailed in Additional file 1. We compared MLIs between groups with analysis of variance.

### Cohort study

#### Study design and participants

We conducted a cohort study to elucidate the role of DEL-1 as a blood biomarker in patients with COPD, including patients from the Korean COPD Subgroup Registry and Subtype Research (KOCOSS) (NCT02800499), an ongoing multicenter cohort study in South Korea. A patient with stable COPD was defined as a person with the post-bronchodilator FEV1/vital capacity (VC) < 0.70 who did not acute experience within 1 month. Those patients who were not followed up for at least 12 months, or who had no blood sample collected at baseline were excluded. Non-COPD participants were randomly selected from the Controls for Respiratory Diseases cohort (NCT03120481), which enrolled those who underwent health screening and consented to participate in the cohort. Inclusion criteria for the non-COPD group were as follows: pre-bronchodilator FEV1/FVC ≥ 0.7, and no history of chronic respiratory diseases. Never smokers and ever smokers were randomly included in a 1:1 ratio. Patients with insufficient blood samples were excluded. Written informed consent was obtained from all participants. The study was approved by the institutional review board of Seoul National University Hospital, Seoul, Korea (IRB no: 2007-029-1139).

#### Measurements of DEL-1 and other variables

For the COPD group, baseline characteristics including demographic factors, smoking history, acute exacerbation history in the past year, modified Medical Research Council (mMRC) grade, 6-min walk distance (6MWD), pulmonary function testing, and laboratory and radiologic findings were evaluated. The history of acute exacerbation and drug treatment were evaluated based on self-reported questionnaire by the study coordinator at 6 months and 12 months, and then annually. An acute exacerbation is defined as a condition that any worsening of cough, sputum, or dyspnea requires additional therapy with systemic corticosteroids or antibiotics, or hospitalization. For the non-COPD group, baseline demographic factors, smoking history, and pulmonary function testing were evaluated.

#### Plasma DEL-1 measurement

DEL-1 levels in humans plasma samples were measured using a commercially available humans EDIL3 DuoSet enzyme-linked immunosorbent assay (ELISA) kit (R&D System, Minneapolis, MN) according to the manufacturer’s instructions.

### Statistical analysis

First, we compared the plasma concentration of DEL-1 between the COPD group and non-COPD group by a linear regression analysis adjusted by age and sex. If measurements were right-skewed, a log transformation was applied. Second, we investigated the association between plasma DEL-1 levels and outcomes in the COPD group. The main outcome was acute exacerbation rates, and a negative binomial regression model adjusted for confounders, including age, sex, acute exacerbation history in the past year, baseline post-bronchodilator FEV1 (% predicted), treatment drugs [inhaled corticosteroids (ICS), long-acting β2-agonists (LABA), long-acting muscarinic agents (LAMA), phosphodiesterase-4 (PDE4) inhibitor] were used, and incidence rate ratios (IRR) with 95% confidence intervals (CIs) were estimated. All statistical analyses were performed using Stata 14.2 (StataCorp, College Station, TX, USA). A *P*-value < 0.05 was considered statistically significant.

## Results

### In vivo study

#### DEL-1 deficiency resulted in emphysema development and augmented CSE-induced emphysema in mice

**Fig. 1:**
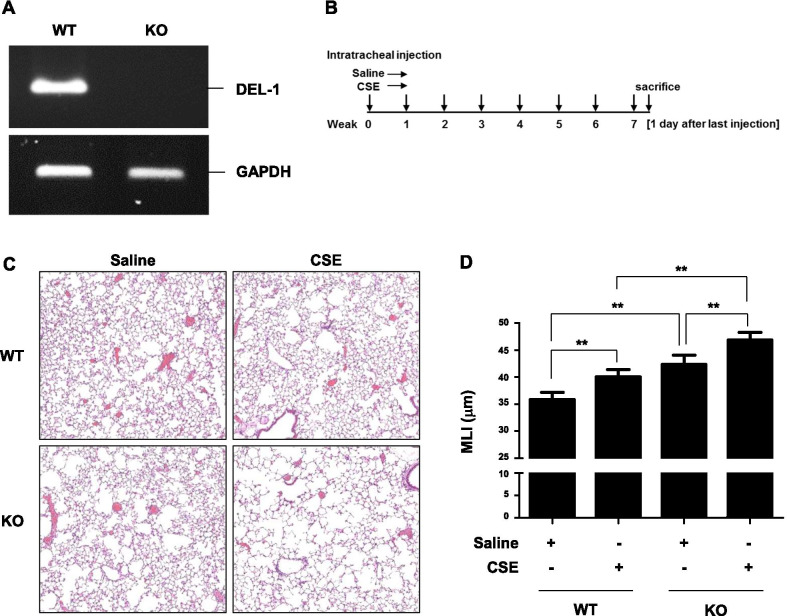
DEL-1 deficiency developed emphysema and augmented cigarette smoke extract (CSE)-induced emphysema in mice. **A** RT-PCR for DEL-1 and GAPDH in lung tissues from WT and DEL-1 KO C57BL/6 mice. **B** WT and DEL-1 KO mice were treated with saline or CSE as described in “Methods” (WT saline; n = 8, WT CSE; n = 8, KO saline; n = 5, KO CSE; n = 8). **C** Lung tissue sections were stained with H&E. Representative photographs of lungs. **D** MLIs are presented as mean ± S.E. (WT saline vs WT CSE, *P* = 0.028; WT saline vs KO saline, *P* = 0.003; KO saline vs KO CSE, *P* = 0.037; WT CSE vs KO CSE, *P* < 0.001) *DEL-1* developmental endothelial locus-1, *RT-PCR*  reverse transcriptase polymerase chain reaction, *GAPDH*  glyceraldehyde 3-phosphate dehydrogenase, *WT*  wild-type, *KO*  knockout, *H&E*  hematoxylin and eosin, *MLI*  mean linear intercept

We first evaluated the role of DEL-1 in the development of emphysema in mice following CSE treatment. DEL-1 KO was confirmed by RT-PCR (Fig. 1A). C57BL/6 WT and DEL-1 KO mice were intratracheally administered saline or CSE as described in the Methods section (Fig. 1B), and the severity of emphysema was determined by measuring MLI. DEL-1 KO mice demonstrated a higher MLI than WT mice, indicating that DEL-1 KO induced emphysema. CSE induced lung parenchymal destruction and led to emphysema, and DEL-1 KO augmented CSE-induced emphysema (Fig. 1C, D).

### Clinical study

#### Characteristics of the study population

**Fig. 2:**
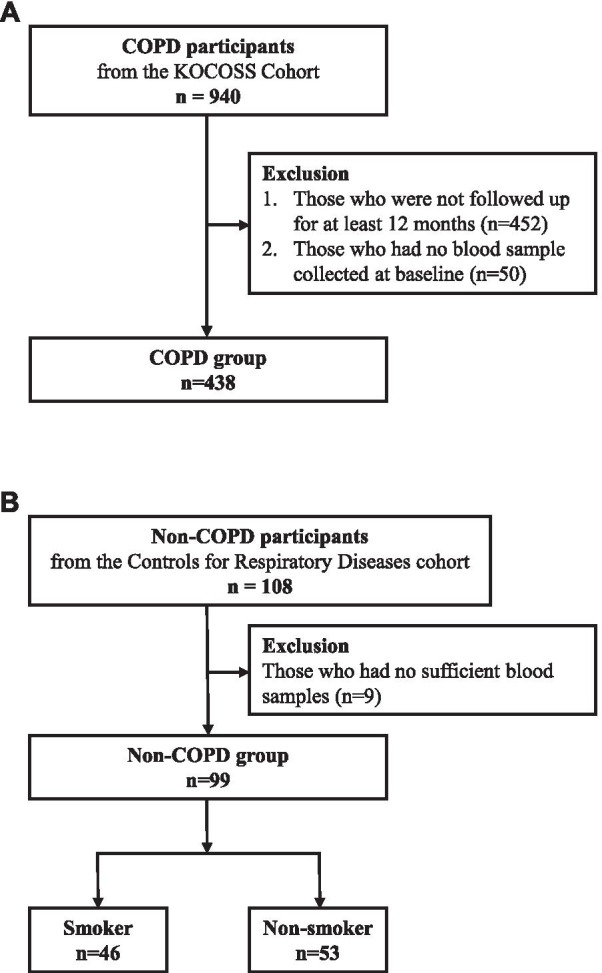
Flowchart for including COPD participants and non-COPD participants. **A** Flowchart of including COPD patients. **B** Flowchart of including non-COPD participants

In total, 940 patients with COPD were eligible from the KOCOSS cohort. Among them, 452 participants who were not followed up for at least 12 months and 50 participants who had no blood sample collected at baseline were excluded. Finally, 438 COPD participants were categorized into the COPD group. Additionally, 108 non-COPD participants were initially recruited, and 9 participants without blood samples were excluded. Overall, 99 non-COPD participants were categorized into the non-COPD group (Fig. 2). The baseline characteristics of COPD patients and non-COPD participants are described in Table 1. The non-COPD group was younger, had more females, and presented a better FEV1 than the COPD group. Among the non-COPD group, the ever-smoker group was older with more male participants than the never-smoker group. Each group demonstrated similar pre-bronchodilator FEV1 levels.

**Table 1:** Baseline characteristics of COPD group and non-COPD group

Characteristics	COPD	Non-COPD			*P*-value
	(n=438)	Total (n=99)	Ever smoker (n=46)	Never smoker (n=53)	
Age (year)	69.0 ± 7.6	60.5 ± 4.8	61.4 ± 4.9	59.8 ± 4.6	*p* < 0.001
Sex (male)	394 (90.2%)	56 (56.6%)	44 (95.7%)	12 (22.6%)	*p* < 0.001
Pack-year	39.0 [24.0;50.0]	0.0 [0.0;17.9]	20.0 [10.0;34.5]	0.0 [0.0; 0.0]	*p* < 0.001
Current smoker	107 (24.4%)	8 (8.1%)			*p* < 0.001
Former smoker	278 (63.5%)	38 (38.4%)			*p* < 0.001
Never smoker	46 (10.5%)	53 (53.5%)			*p* < 0.001
Unknown	7 (1.6%)				
Pre-bronchodilator FEV1(% predicted)	65.0 [51.0;77.0]	102.0 [93.5;111.0]	101.0 [94.0;111.0]	104.0 [93.0;111.0]	*p* < 0.001
Post-bronchodilator FEV1(% predicted)	68.0 [54.0;80.0]				
Exacerbation history in past year	57.0 (13.0%)				
mMRC	1.14±0.82				
6MWD (meter)	439.0 [370.0;490.8]				
Lab finding					
WBC (×10³/mm³)	6.7 [5.8; 8.3]	4.8 [ 4.0;5.9]	5.2 [ 4.2; 6.0]	4.8 [ 3.9;5.6]	*p* < 0.001
Neutrophil (%)	58.4 [51.4;64.2]	55 [48.1;61.6]	57.1 [49.2;60.6]	53.3 [46.8;61.9]	*p* = 0.008
Lymphocyte (%)	29.2 [23.4;35.7]	34.3 [28.6;40.4]	33.6 [28.8;38.5]	35.6 [28.6;41.7]	*p* < 0.001
CRP (mg/dL)	0.8 [0.4; 1.9]				
Fibrinogen (mg/dL)	304.5 [256.5;347.0]				

Data are presented as mean ± standard deviation, median [interquartile range], or frequency with percentage

*mMRC*  modified Medical Round Council, *6WMD*  six-minute walk distance, *WBC*  white blood cell, *CRP*  c-reactive protein

#### Patients with COPD had lower plasma levels of DEL-1 compared with non-COPD never smokers

**Fig. 3:**
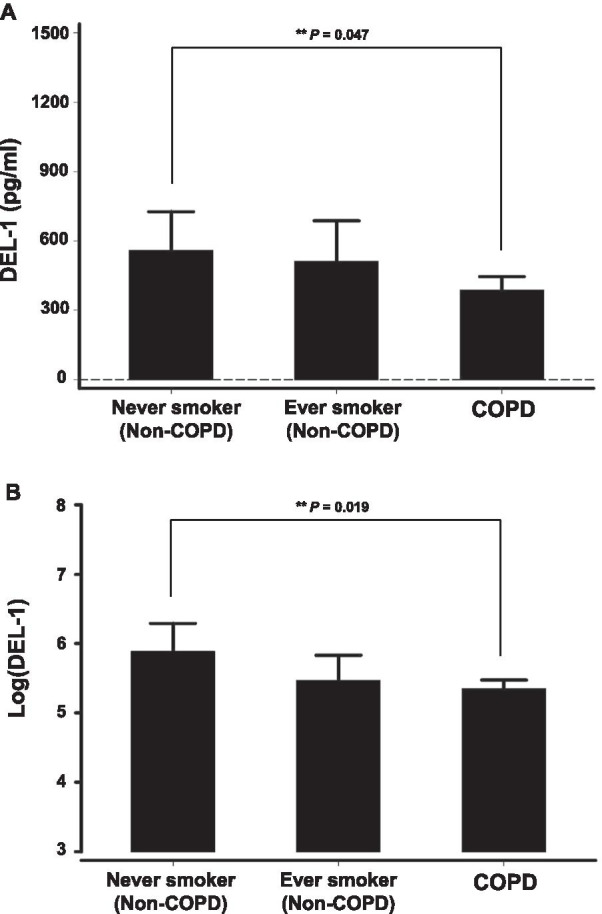
COPD patients had a plasma DEL-1 level lower than non-COPD never smokers. **A** Plasma levels of DEL-1 in COPD group, non-COPD ever smokers and non-COPD never smokers. (**age, sex-adjusted *P*-values: COPD vs total non-COPD,* P* = 0.030; COPD vs never smokers, *P* = 0.047; ever smokers vs never smokers, *P* = 0.44; COPD vs ever smokers, *P* = 0.24). **B** Plasma levels of Log(DEL-1) in COPD group and, non-COPD ever smokers and non-COPD never smokers. (**age, sex-adjusted *P*-values: COPD vs total non-COPD, *P* = 0.040; COPD vs never smokers, *P* = 0.019; ever smokers vs never smokers, *P* = 0.13; COPD vs ever smokers, *P* = 0.58)

Patients with COPD presented lower levels of DEL-1 than non-COPD participants (385.9 ± 523.0 vs. 535.4 ± 903 pg/mL; *P* = 0.03). Furthermore, patients with COPD revealed lower levels of DEL-1 when compared with non-COPD never smokers, when age and sex were adjusted (*P* = 0.047) (Fig. 3A). Log transformation of the DEL-1 value was performed as the distribution of measured DEL-1 was right-skewed. After log transformation of DEL-1, patients with COPD similarly showed lower levels of Log(DEL-1) than those without COPD (5.4 ± 1.2 vs. 5.6 ± 1.2 pg/mL; *P* = 0.04). After adjusting for age and sex, patients with COPD also presented lower levels of Log(DEL-1) than non-COPD never smokers (*P* = 0.019) (Fig. 3B).

#### Low plasma DEL-1 level was associated with increased risk of subsequent acute exacerbation in patients with COPD.

**Fig. 4:**
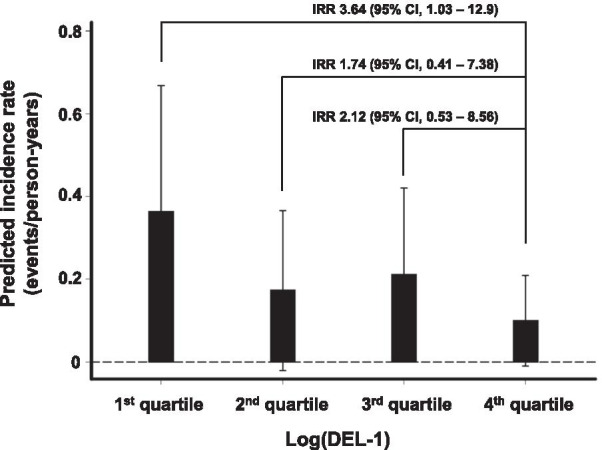
A low plasma DEL-1 level was associated with increased risk of subsequent exacerbation in COPD patients

During a mean 1.2** ± **0.3 years follow-up period, 67 acute exacerbation events were reported among patients with COPD (incidence rate, 0.15 ± 0.75 events per person-years). The lowest quartile of the Log(DEL-1) group (incidence rate, 0.36 events per person-year; 95% CI, 0.06–0.67) revealed a risk of subsequent acute exacerbations higher than the highest quartile group (incidence rate, 0.10 events per person-year; 95% CI, − 0.01–0.21). On adjusting the covariates, the lowest quartile group presented more than three times increased risk of acute exacerbations during the follow-up period when compared with the highest quartile group (IRR, 3.64; 95% CI, 1.03–12.9) (Fig. 4).

## Discussion

In the present study, our findings demonstrated that the level of plasma DEL-1 in stable COPD patients was lower than that in non-COPD patients, with a low baseline DEL-1 level associated with a higher risk of subsequent acute exacerbation in the latter period in patients with COPD.

Although some patients with COPD do not experience acute exacerbations, other patients frequently do experience this complication. Acute COPD exacerbations reduce a patients’ quality of life [3], accelerate disease progression [4], and considerably increase healthcare costs [34]. As several patients with COPD do not report their acute exacerbation events to healthcare providers [35], the rate of acute exacerbation may be underestimated [36]. Thus, if a reliable and easily measurable biomarker to predict the subsequent incidence of acute exacerbation is identified, it would be extremely valuable in the management of patients with COPD.

Researchers have extensively explored biomarkers predicting acute COPD exacerbations and identified some biomarkers. The biomarkers of interest were systemic inflammatory biomarkers. Reportedly, ECLIPSE cohort researchers have measured six systemic inflammatory biomarkers in the peripheral blood of participants, including white blood cell count and CRP, IL-6, IL-8, fibrinogen, and TNF-α levels. They observed that those who presented persistent systemic inflammation were at a higher risk of all-cause mortality and more frequent acute exacerbations when compared with non-inflamed participants [12]. In participants of the Copenhagen City Heart Study, a serum CRP level > 3 mg/L was associated with an increased risk of hospitalization and death due to COPD [37]*.* Furthermore, a high plasma fibrinogen level ≥ 350 mg/dL was associated with an increased risk of hospitalization owing to acute COPD exacerbation within 12 months and all-cause mortality within 36 months in pooled data from Atherosclerosis Risk in Communities Study (ARIC), Cardiovascular Health Study (CHS), and Evaluation of COPD longitudinally to Identify Predictive Surrogate Endpoints (ECLIPSE) study [38]. However, these systemic inflammatory biomarkers have limitations. The concentration of a blood biomarker might be influenced by prior acute exacerbation events that were similar to overwhelming systemic inflammation [36]*.* Additionally, although acute exacerbations originate in the airways, the relationship between airway and systemic inflammation remains unclear considering the poor correlation between sputum and blood biomarkers [13, 14]. Moreover, there are no drug candidates that target the direct reduction of systemic inflammatory biomarkers [39], although studies have reported that fibrinogen is reduced by p38 mitogen-activated protein kinase inhibitors [40]*.* In addition to systemic inflammation, other biomarkers have been identified, reportedly related to subsequent acute exacerbation risk. In the ECLIPSE cohort, a high serum surfactant protein D (SP-D) level > 175.4 ng/mL (greater than the 95th percentile of nonsmokers) has been associated with an increased risk of acute exacerbation within 12 months [15]*.* However, one study has revealed that SP-D levels are reportedly reduced in the bronchoalveolar lavage fluid of patients with COPD [17], which questions the usefulness of SP-D. Another study has reported that an increased plasma level of CC-16 was reportedly associated with a decreased number of subsequent acute exacerbations during 6 months of follow-up; however, this study included only a small number (n = 38) of participants [16]*.* Considering that these systemic biomarkers have limitations, efforts to identify new reliable biomarkers are warranted.

To our knowledge, the present study is the first to suggest DEL-1 as a novel blood biomarker for predicting subsequent acute exacerbations in patients with COPD. Although our study was unable to identify how DEL-1 decreases the risk of acute COPD exacerbations, several explanations could be considered. First, DEL-1 has anti-inflammatory properties. DEL-1 inhibits neutrophil recruitment by interrupting the binding of lymphocyte function-associated antigen 1 (LFA-1) and intercellular adhesion molecule-1 (ICAM-1) [19]*.* Furthermore, DEL-1 enhances macrophage efferocytosis and clearance of inflammation [20, 21]*.* Airway and systemic inflammation are principal mechanisms of acute exacerbation of COPD, and DEL-1 can protect from acute exacerbations by decreasing inflammation [22]*.* Second, DEL-1 may protect against cardiovascular events by inhibiting inflammation [27]. Cardiovascular diseases increase the risk of acute COPD exacerbations [41]; additionally, acute COPD exacerbations increase the risk of subsequent cardiovascular events [42] that could worsen patient symptoms. Thus, DEL-1 could contribute to cardiovascular stability, leading to a decreased risk of COPD worsening.

Moreover, our study revealed that lower level of DEL-1 could contribute to the pathogenesis of COPD. DEL-1 knockout resulted in the development and augmentation of emphysema in a mouse model. Furthermore, patients with COPD presented plasma DEL-1 levels lower than non-COPD controls (385.9 ± 523.0 vs 535.4 ± 903.2 pg/mL; *P* = 0.03; Log(DEL-1), 5.4 ± 1.2 vs 5.6 ± 1.2 pg/mL; *P* = 0.04), especially non-COPD never smokers. (log (DEL-1), *P* = 0.019). As discussed above, DEL-1 demonstrates anti-inflammatory effects by inhibiting neutrophil recruitment, promoting macrophage efferocytosis, and clearance of inflammation. Persistent inflammation, in which neutrophils and macrophages are known contributors, has been regarded as the key factor in the pathogenesis of COPD [43]. Additionally, DEL-1 has inhibitory effects on endothelial cell apoptosis [23], along with anti-fibrotic [24, 25], anti-aging [26], and anti-protease effects [27, 28]*,* suggesting that a reduced DEL-1 level can be related to the pathogenesis of COPD.

We acknowledge limitations. First, non-COPD participants were enrolled from another cohort, and were not matched to COPD patients. It could raise a concern for selection bias and confounding effects, although we adjusted age and sex as covariates for the comparison between groups. Second, although our cohort enrolled stable COPD patients who were not in acute exacerbation within one month, there might some participants, even though the number may be very small, who were not ‘stable’ at baseline considering a full recovery from acute exacerbation may not occur by months in a small number of patients [44]. A remaining acute exacerbation could affect the DEL-1 level. Third, DEL-1 was measured only at baseline in our study. As repeated measurement could provide additional clinical implications, further studies are needed. Fourth, there could be another factors affecting DEL-1 levels. We did not perform subgroup analysis according to comorbidities, although it has been reported that DEL-1 could be decreased in some comorbid diseases such as inflammatory disorders [45]. Fifth, we did not evaluate the association between DEL-1 level and other variables including lung function and airway inflammation that are linked to COPD pathogenesis. To validate the role of DEL-1, further studies are needed.

## Conclusion

In conclusion, we demonstrated that low plasma DEL-1 levels are associated with COPD diagnosis and an increased risk of subsequent acute COPD exacerbation. Our results suggest that DEL-1 could be a good biomarker in patients with COPD. Considering these beneficial effects on COPD, DEL-1 can be further researched in the prevention and treatment of COPD.

## Supplementary Information

**Additional file 1**. Supporting information on methods.

## Data Availability

The data supporting our findings are available from the corresponding author on reasonable request.

## Abbreviations

COPD: Chronic obstructive pulmonary disease
DEL-1: Developmental endothelial locus-1
CSE: Cigarette smoke extract
WT: Wild-type
KO: Knockout
MLI: Mean linear intercept
FEV1: Forced expiratory volume in one second
IRR: Incidence rate ratios
CI: Confidence interval
