# Supplementary material for: Developmental endothelial locus-1 as a potential biomarker for the incidence of acute exacerbation in patients with chronic obstructive pulmonary disease
Source: Respir Res. 2021 Nov 20;22:297. doi: 10.1186/s12931-021-01878-7 (PMC8605521; doi:10.1186/s12931-021-01878-7)
Supplement: Supplementary file 1 — Additional file 1. Supporting information on methods. [file 12931_2021_1878_MOESM1_ESM.docx]

**Additional Information**

**Methods**

**CSE preparation**

Commercial cigarettes (THIS; 84 mm long with a diameter of 8 mm, purchased from Korea Tomorrow & Global Corp.) were smoked continuously using a bottle system connected to a vacuum machine. The smoke from 20 cigarettes was bubbled in 60 mL of PBS (GIBCO). The large insoluble particles contained in the resulting suspension were removed by filtering the solution through a 0.22 μm filter.

# Measurement of Emphysema

# Mouse lungs were fixed with 4% neutral buffered paraformaldehyde. Fixed lung was dehydrated, embedded with paraffin, sectioned, and stained with hematoxylin and eosin (H&E). Emphysema was quantified by measuring the mean linear intercept (MLI). Four randomly selected x100 fields per specimen were photographed in a blinded manner. The MLI was measured by placing four 1000 μm lines over each field. The total length of each line divided by the number of alveolar intercepts gives the average distance. The non-parenchymal area was not included
